# Volvulus and Torsion of Various Organs Requiring Emergency Surgery: A Case Series

**DOI:** 10.1002/ccr3.70930

**Published:** 2025-09-24

**Authors:** Halil İbrahim Altunbulak, Bilal Altunbulak, Ahmet Yasir Altunbulak

**Affiliations:** ^1^ Department of Radiology Hacettepe University Ankara Türkiye; ^2^ Department of Radiology State Hospital of Tatvan Bitlis Türkiye; ^3^ Department of Radiology Bilkent City Hospital of Ankara Ankara Türkiye; ^4^ Department of Radiology State Hospital of Batman Batman Türkiye

**Keywords:** emergency, life‐threatening, surgery, torsion, volvulus

## Abstract

Volvulus and torsion are life‐threatening emergencies requiring prompt diagnosis and surgical intervention. This study presents cases gallbladder torsion, lung torsion, mesenteroaxial gastric volvulus. Each case emphasizes the role of advanced imaging, particularly computed tomography (CT), in identifying these conditions. Despite timely surgical management, outcomes vary depending on the degree of ischemia and the patient's overall condition. Early recognition and intervention are crucial to prevent complications such as necrosis and multiorgan failure. Increased awareness and rapid surgical treatment can significantly improve patient outcomes.

## 1 Introduction

Volvulus and torsion are generally life‐threatening and morbid conditions that require emergency surgical intervention. Our aim is to highlight the critical urgency of managing volvulus and torsion.

## 2 Case Descriptions

All patients provided written informed consent prior to their inclusion in the study.

### 2.1 Key Clinical Messages for Gallbladder Torsion

Gallbladder torsion, especially in elderly females, should be considered in cases of acute abdomen with imaging findings of a floating gallbladder and absent gallstones; prompt surgery is essential to prevent fatal outcomes.

#### 2.1.1 Galbladder Torsion

A 90‐year‐old female patient with a history of multiple comorbidities presented to the emergency department with nonspecific abdominal symptoms. Physical examination findings were inconclusive; however, given the suspicion of an acute abdomen, contrast‐enhanced computed tomography (CT) was performed.

**FIGURE 1:**
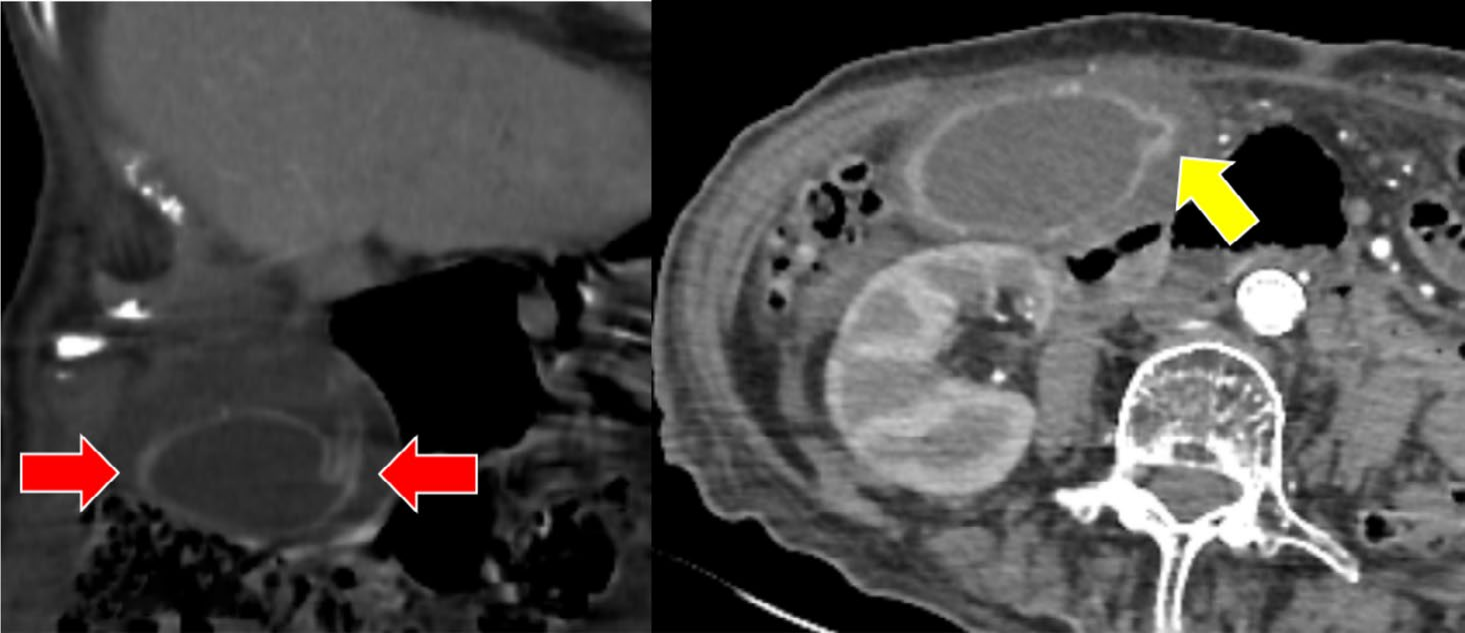
A 90‐year‐old female patient presenting with abdominal distension and pain. The gallbladder appears abnormally positioned, rotated from a vertical to a horizontal orientation outside its normal fossa, surrounded by pericholecystic fluid (red arrows). Additionally, an abrupt caliber change is noted from the gallbladder neck to the cystic duct (yellow arrow). These findings are consistent with gallbladder torsion.

CT imaging revealed a distended gallbladder with pericholecystic fluid and a twisted appearance outside its normal anatomical fossa, suggesting a rotation along its mesentery. The gallbladder was abnormally positioned, with its axis shifted from a vertical to a horizontal orientation. Additionally, an abrupt caliber change at the cystic duct and a beak‐like tapering at the gallbladder neck were noted, consistent with gallbladder torsion. No significant gallstones or biliary duct dilation were observed (Figure 1, Video 1).

**VIDEO 1:** Gallbladder torsion. Video content can be viewed at https://onlinelibrary.wiley.com/doi/10.1002/ccr3.70930.

Given the diagnosis, the patient underwent emergent open cholecystectomy. Intraoperative findings confirmed gallbladder torsion with ischemic changes but no evidence of perforation. Postoperatively, the patient required intensive care unit (ICU) admission due to hemodynamic instability. Despite supportive care, the patient's condition deteriorated, and she unfortunately succumbed to multi‐organ failure during ICU follow‐up.

### 2.2 Key Clinical Messages for Lung Torsion

Lung torsion, though rare, must be promptly recognized on CT when non‐enhancing lobes and twisted bronchovascular pedicles are seen, especially in the presence of massive effusion, to avoid irreversible ischemia.

#### 2.2.1 Lung Torsion

A 79‐year‐old male with a history of multiple comorbidities, including chronic obstructive pulmonary disease (COPD) and hypertension, presented to the emergency department with progressive dyspnea and right‐sided chest pain. Given his age and clinical presentation, potential differential diagnoses included pulmonary thromboembolism, lung malignancy, pneumonia, and heart failure‐related effusion.

**FIGURE 2:**
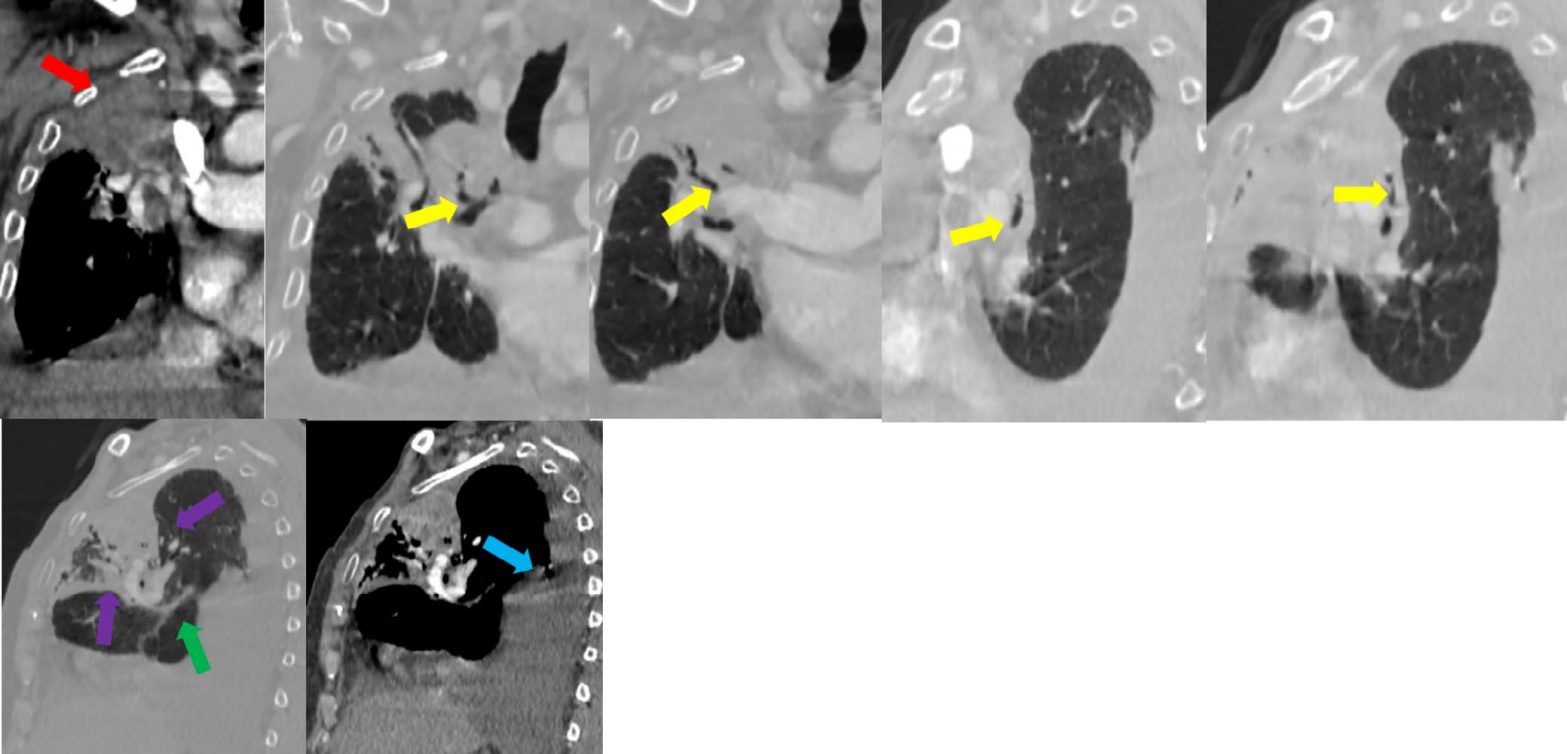
A 79‐year‐old male patient with a non‐enhancing atelectatic region in the right upper lung (red arrow), narrowing of the bronchovascular pedicle associated with the atelectatic lung tissue (yellow arrow), and a curvilinear course of the vascular structures (green arrow). Additionally, distortion of the major fissure (purple arrow) and a massive pleural effusion (blue arrow) are observed. These findings are consistent with lung torsion.

Contrast‐enhanced computed tomography (CT) of the thorax revealed a non‐enhancing, atelectatic right upper and middle lobe with an abrupt narrowing of the bronchovascular pedicle. Additionally, a curvilinear orientation of the bronchovascular structures, distortion of the major fissure, and an associated massive pleural effusion were noted. These findings were highly suggestive of lung torsion, with the effusion likely acting as a predisposing factor by altering the normal anatomic support of the lung lobes (Figure 2, Videos 2A and 2B).

**VIDEO 2A:** Lung torsion on mediastinal window. Video content can be viewed at https://onlinelibrary.wiley.com/doi/10.1002/ccr3.70930.

**VIDEO 2B:** Lung torsion on lung window. Video content can be viewed at https://onlinelibrary.wiley.com/doi/10.1002/ccr3.70930.

Given the diagnosis, the patient was taken to surgery for urgent video‐assisted thoracoscopic surgery (VATS). Intraoperatively, a 360‐degree torsion of the right upper and middle lobes was confirmed. Due to irreversible ischemic changes, an upper‐middle lobectomy was performed. The remaining lung expanded well post‐procedure.

Postoperatively, the patient had an uneventful recovery and was discharged without complications. Follow‐up imaging confirmed satisfactory expansion of the residual lung, with no signs of recurrence or infection.

### 2.3 Key Clinical Messages for Mesenteroaxial Gastric Volvulus

Mesenteroaxial gastric volvulus may present subtly but requires high suspicion when nasogastric intubation fails and CT reveals abnormal antrum position; early laparoscopic detorsion prevents severe gastric complications.

#### 2.3.1 Mezenteroaxial Volvulus of Stomach

A 75‐year‐old male with no known chronic medical conditions presented to the emergency department with persistent retching but without nausea or vomiting. He had no prior history of gastrointestinal disorders or previous abdominal surgeries. On physical examination, he was hemodynamically stable with mild epigastric tenderness but no peritoneal signs.

**FIGURE 3:**
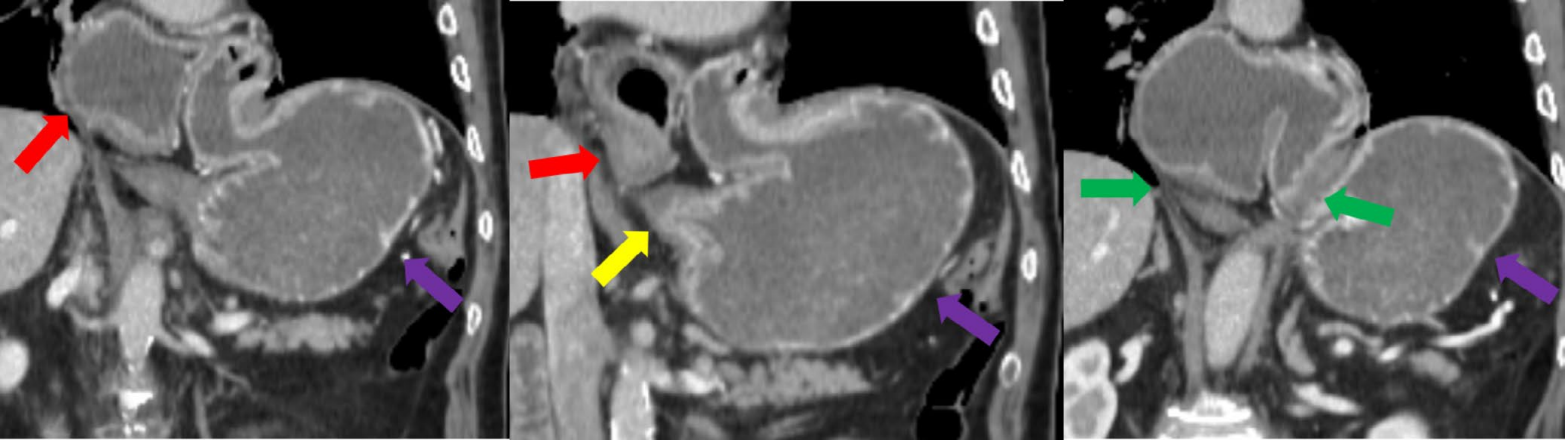
A 75‐year‐old male patient with the antrum and pylorus (red arrow) positioned superior to the gastric fundus (purple arrow), and the antropyloric region located superior to the gastroesophageal junction (yellow arrow). A large, wide‐necked hiatal hernia (green arrow) is also observed. These findings are consistent with mesenteroaxial gastric volvulus. No signs of ischemia, perforation, or gastric outlet obstruction were detected.

A nasogastric (NG) tube was attempted multiple times but could not be advanced beyond the gastroesophageal junction, raising suspicion of gastric volvulus. Contrast‐enhanced computed tomography (CT) of the abdomen was performed, revealing classic imaging findings of mesenteroaxial gastric volvulus. The antrum and pylorus were found to be abnormally positioned superior to the gastroesophageal junction, with a large, wide‐necked hiatal hernia. The stomach was rotated around its short axis, but no signs of gastric wall ischemia, perforation, or gastric outlet obstruction were observed (Figure 3, Video 3).

**VIDEO 3:** Mesenteroaxial volvulus. Video content can be viewed at https://onlinelibrary.wiley.com/doi/10.1002/ccr3.70930.

Given the diagnosis, the patient was taken to the operating room for laparoscopic intervention. Intraoperatively, a complete mesenteroaxial volvulus was confirmed. Laparoscopic detorsion was successfully performed, followed by gastropexy to prevent recurrence. The patient tolerated the procedure well, and no postoperative complications occurred. He resumed oral intake without difficulty and was discharged uneventfully.

## 3 Discussion

### 3.1 Galbbladder Torsion

Gallbladder torsion is a rare condition that requires emergency surgical intervention to prevent mortality and morbidity. It predominantly affects elderly women [1]. A floating gallbladder with a long cystic duct predisposes the organ to torsion [2]. Due to the absence of specific symptoms and the overlap of clinical presentation with acute cholecystitis, the diagnostic process can be challenging.

Gallbladder torsion should be suspected in cases where computed tomography (CT) and ultrasound imaging show an absence of gallstones within the gallbladder lumen, displacement of the gallbladder from its usual anatomical location, and normal liver function tests [3]. Given the risk of complications such as perforation, early diagnosis and prompt surgical management with cholecystectomy are essential [1].

### 3.2 Lung Torsion

Lung torsion is a rare but potentially life‐threatening condition that occurs when a lung lobe or the entire lung rotates around its bronchovascular pedicle. It most commonly affects the right middle lobe, followed by the right upper lobe [4].

Due to the nonspecific nature of its clinical presentation, lung torsion should be suspected, particularly in the postoperative setting. In diagnosis, although direct signs such as twisting of the pedicle can occasionally be observed on computed tomography (CT), indirect signs are more common. These include loss of normal parenchymal contrast enhancement, atelectasis of the torsed lung, and abnormal positioning of the fissure [5].

Predisposing factors for lung torsion include lung resection [6, 7], large‐volume thoracentesis, the presence of a mass, and massive pleural effusion [8]; however, it can also occur spontaneously. Once diagnosed, emergency surgical intervention is required to prevent severe complications [6].

### 3.3 Mezenteroaxial Volvulus of Stomach

Gastric volvulus is an abnormal rotation of the stomach, occurring either along the organoaxial or mesenteroaxial axis [9, 10]. While the organoaxial type is more common, the mesenteroaxial type is rare and is typically seen in children and adults in their 50s [11]. This condition is often associated with hiatal hernia and its surgical repair [12, 13].

Patients usually present with nonspecific symptoms such as abdominal pain, nausea, and vomiting. However, these symptoms may also indicate serious complications such as ischemia or perforation. If left untreated, gastric volvulus can lead to severe conditions, including ischemia, perforation, and gastric outlet obstruction [14].

Diagnosis is primarily made using computed tomography (CT), where the gastric antrum and pylorus are seen displaced cranially relative to the gastric fundus [15]. Treatment options include laparoscopic detorsion [16], particularly in elderly patients with comorbidities and high surgical risk. In cases of detorsion resistance, open gastropexy or Roux‐en‐Y gastric bypass surgery may be required [10].

## 4 Conclusion

Volvulus and torsion of various organs are life‐threatening conditions that require timely recognition and urgent surgical intervention. Delayed diagnosis can lead to ischemia, necrosis, and multiorgan failure, significantly increasing morbidity and mortality. Advanced imaging modalities, particularly computed tomography (CT), play a crucial role in early and accurate diagnosis. Prompt surgical management, tailored to the specific organ involved, is essential to improve patient outcomes. Increased awareness among clinicians and radiologists is critical for early detection and intervention, ultimately reducing complications and improving survival rates.

## Author Contributions

**Halil İbrahim Altunbulak:** conceptualization, data curation, writing – original draft, writing – review and editing. **Bilal Altunbulak:** conceptualization, data curation. **Ahmet Yasir Altunbulak:** supervision, validation.

## Consent

All patients provided written informed consent prior to their inclusion in the study.

## Conflicts of Interest

The authors declare no conflicts of interest.

## Data Availability

All data generated or analyzed during this study are included in this published article. Further inquiries can be directed to the corresponding authors.
